# Transcriptional control of GABAergic neuronal subtype identity in the thalamus

**DOI:** 10.1186/1749-8104-9-14

**Published:** 2014-06-15

**Authors:** Katherine Sellers, Verena Zyka, Andrew G Lumsden, Alessio Delogu

**Affiliations:** 1 MRC Centre for Developmental Neurobiology, King’s College London, London SE1 1UL, UK; 2 Present address: Department of Neuroscience, Institute of Psychiatry, King’s College London, London SE5 9NU, UK

**Keywords:** *Helt*, *Dlx2*, *Sox14*, GABAergic thalamus, Neurogenesis, Diencephalon

## Abstract

**Background:**

The thalamus is often defined as the ‘gateway to consciousness’, a feature that is supported by the specific connectivity and electrophysiological properties of its neurons. Inhibitory GABAergic neurons are required for the dynamic gating of information passing through the thalamus. The high degree of heterogeneity among thalamic GABA neurons suggests that, during embryonic development, alternative differentiation programmes exist to guide the acquisition of inhibitory neuron subtype identity.

**Results:**

Taking advantage of the accessibility of the developing chick embryo, we have used *in ovo* manipulations of gene expression to test the role of candidate transcription factors in controlling GABAergic neuronal subtype identity in the developing thalamus.

**Conclusions:**

In this study, we describe two alternative differentiation programmes for GABAergic neurogenesis in the thalamus and identify Helt and Dlx2 as key transcription factors that are sufficient to direct neuronal progenitors along a specific differentiation pathway at the expense of alternative lineage choices. Furthermore, we identify *Calb2*, a gene encoding for the GABA subtype marker calretinin as a target of the transcription factor Sox14. This work is a step forward in our understanding of how GABA neuron diversity in the thalamus is achieved during development and will help future investigation of the molecular mechanisms that lead up to the acquisition of different synaptic targets and electrophysiological features of mature thalamic inhibitory neurons.

## Background

The thalamus plays a crucial function in ensuring faithful transfer of sensory information to the cortex. Excitatory, glutamatergic relay neurons constitute the largest neuronal type in the thalamus. However, thalamic function is not restricted simply to relaying information to and from the cortex: the thalamus can highlight certain inputs and suppress others, a feature that is evident during deep sleep when coordinated oscillations in the thalamo-cortical system suppress the ascending flow of peripheral and sensory inputs [1], or in pathological conditions such as schizophrenia [2] and absence epilepsy [3] when, respectively, hallucinations and temporary loss of consciousness can occur. The ability to modulate the flow of information is supported by a second abundant neuronal type, the inhibitory GABA neurons of the reticular nucleus of the thalamus (TRN). TRN neurons are defined by the expression of the *parvalbumin* gene [4], their fast spiking action and specific connectivity, which is strictly confined within the thalamus. While TRN neurons are a near homogeneous population, the remaining GABA inhibitory neurons in the thalamus (nonTRN GABA thalamus) differ from those of the TRN in their anatomical position, morphology, connectivity, and molecular profile, and consequently also function. NonTRN GABA neurons can be grouped into two large categories: local interneurons active within the thalamus and projecting neurons with extra-thalamic targets. The TRN neurons derive from GABAergic progenitors in the embryonic prethalamic (pTh) compartment [5]. Whilst it was long thought that all thalamic inhibitory neurons originate in the pTh, it is now clear that the thalamus proper contains a resident population of neuronal progenitors fated to become inhibitory. Most nonTRN GABA neurons develop from a progenitor type in the rostral part of the thalamus (rTh) [6,7].

Acquisition of cell lineage identity in the developing thalamus is regulated by the activity of a local organizer, the zona limitans intrathalamica (zli) acting via secretion of the morphogen molecules Shh, Wnts and FGFs [8-12]. Our and other groups have shown that different developmentally regulated transcription factors are induced by zli signaling on both sides of the organizer [8-10,12-17]. We have recently reported that in the pTh the pro-GABAergic transcription factors Dlx1/2, which are required for development of the TRN [5], suppress the rTh nonTRN GABA differentiation programme [6]. This finding is consistent with the recent discovery that the rTh *Gata2* gene exerts a similar and reciprocal function, by suppressing pTh TRN fate [18]. Both the *Dlx1*/*2* and *Gata2/3* loss-of-function data suggest that on each side of the diencephalic organizer zli, two alternative developmental programmes lead to the formation of GABAergic neurons. Here, we have investigated further some of the transcriptional events that define the differentiation trajectories of the two main subtypes of thalamic GABAergic neurons using the chick embryo system. Firstly, we demonstrate that the transcription factor Helt is sufficient to drive differentiation along the nonTRN GABA programme within the thalamus. Second, we confirm and provide novel evidence that Dlx2 acts as a lineage specification factor within the pTh to induce TRN fate at the expenses of the nonTRN one. Third, we report that Sox14, a post mitotic transcription factor expressed in the rTh, is necessary and sufficient to confer some nonTRN GABA neuron subtype features. In conclusion, this work provides new experimental evidence towards understanding the transcriptional programmes driving the acquisition of GABAergic subtype diversity in the thalamus.

## Results and discussion

### Asymmetric GABAergic neurogenesis at the Th/pTh border

**Figure 1:**
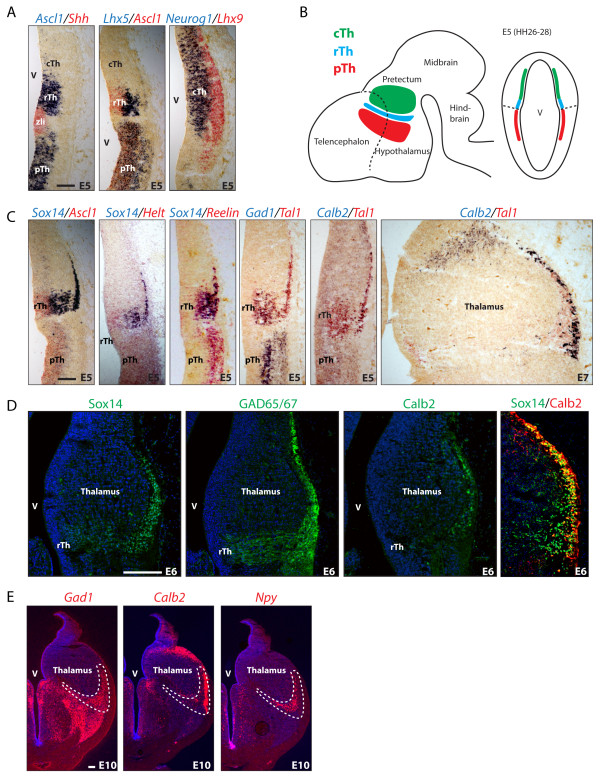
**GABA progenitor domains in the chick thalamus.** Progenitor territories with a GABAergic fate in the thalamus and pTh are visualized by expression of *Ascl1* and *Lhx5* and are present on both sides of the regional organizer zli, labeled by *Shh* expression. Thalamic progenitors positioned further away from the zli acquire a glutamatergic fate and express *Neurog1* and *Lhx9***(A)**. Schematic drawing illustrating the position of prethalamic (pTh), rostral thalamic (rTh) and caudal thalamic (cTh) progenitor domains in lateral and coronal views of the developing chick brain **(B)**. *Helt*, *Tal1* and *Sox14* are expressed in rTh progenitors and precursors. Their expression overlaps with GABAergic markers *Gad1* and *Reelin*. From the fifth day of incubation (E5), rTh GABA derivatives express the interneuron subtype marker and calcium binding protein *Calretinin* (*Calb2*) **(C)**. E6 rTh neurons can be visualized by immunodetection of the GABA-synthesizing enzymes GAD65/67. The calcium binding protein Calb2 is present and co-expressed with the transcription factor Sox14 in a subset of rTh neurons **(D)**. Position of rTh progenitors in the perirotundic area of the chick thalamus by day E10 of embryonic development. The *Calb2*-expressing neurons appear to segregate from *Npy*-expressing neurons within the perirotundic area and may represent two subtypes within the rTh pool. Both cell groups are labeled by the panGABAergic marker *Gad1***(E)**. Scale bars: 100 μm.

Similar to the mouse, the chick thalamus expresses the panGABAergic marker *Gad1* in the pTh and rTh (Figure 1C). Mirroring *Gad1* expression are GABA-related transcription factors *Ascl1* and *Lhx5* (Figure 1A). Both progenitor domains, the pTh and rTh, also express *Reelin* (Figure 1C). We recently defined the rTh in the mouse by the sequential expression of *Helt*, *Tal1* and *Sox14* transcription factor genes [6]; a similar cascade of homologous genes defines the rTh avian equivalent (Figure 1C). In the chick, post mitotic rTh neurons express the GABA subtype marker *Calb2* (Figure 1C, D, E) and, in common with the mouse, the *Npy* GABA subtype marker. Later in embryonic development, *Sox14* positive rTh derivatives appear to segregate in a lateral *Calb2* positive domain and a medial *Npy* positive domain (Figure 1E). The larger progenitor domain in the caudal thalamus (cTh) (Figure 1B) expresses *Neurog2* and *Lhx9* (Figure 1A) and acquires a glutamatergic fate, as described already in other model systems: the zebrafish [14] and the mouse [7].

### Helt is sufficient to induce rostral thalamic identity in the thalamus

**Figure 2:**
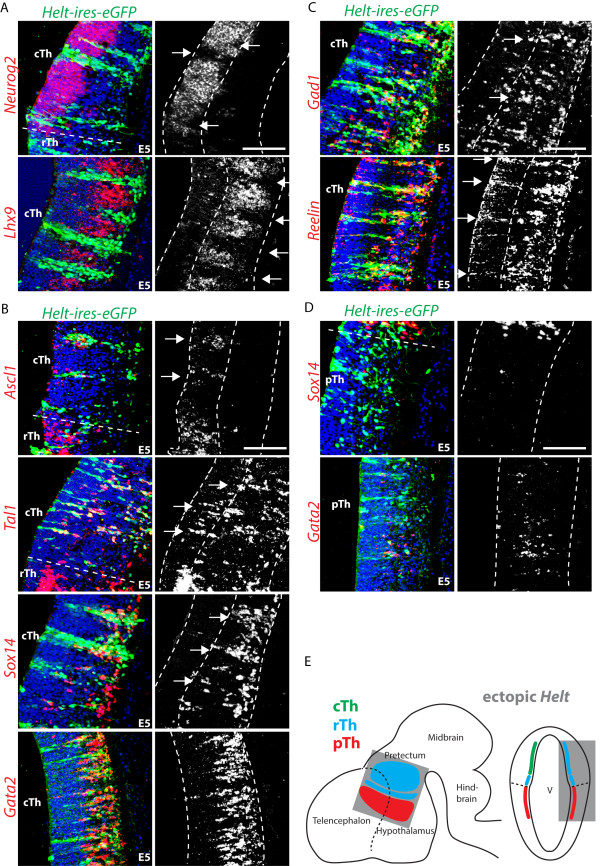
***Helt *expression is sufficient for rostral thalamic (rTh) subtype specification.** Ectopic expression of the rTh transcription factor Helt in the caudal thalamic (cTh) glutamatergic progenitor domain at E2.5, suppresses *Neurog2* and *Lhx9* expression, indicative of a loss of glutamatergic progenitor identity **(A)**. cTh progenitors expressing ectopic *Helt*, acquire expression of all rTh markers tested: proliferative zone (*Ascl1*) and postmitotic compartment (*Gata2*, *Tal1* and *Sox14*) **(B)**. PanGABAergic markers are also induced (*Gad1* and *Reelin*) **(C)**. Ectopic *Helt* expression in the prethalamic (pTh) GABA progenitor domain fails to induce rTh subtype identity, suggesting that other co-factors may be required **(D)**. Schematic drawing summarizing the effect of ectopic *Helt* expression in the three progenitor domains of the thalamus: cTh, rTh and pTh **(E)**. Scale bars: 100 μm.

While gain- and loss-of-function experiments have revealed an important function for *Helt* in the embryonic mouse midbrain [19,20]; *Helt*’s role in the developing diencephalon remains more ambiguous. *Helt* is required for GABAergic differentiation in the pretectum, but not in the rTh [6] and ectopic expression of the gene was not sufficient to induce the GABA fate in the mouse diencephalon, possibly due to the reported early lethality of the transgenic strategy used [21]. We took advantage of the accessibility of the chick embryo to perform *in ovo* electroporation of a constitutively active *Helt* expression construct in the diencephalon. Ectopic *Helt* expression in the thalamus prior to the onset of neurogenesis (E2.5) suppresses the glutamatergic fate, indicated by the lack of *Neurog2* and *Lhx9* expression by E5 (Figure 2A) and concomitant differentiation towards a rTh subtype that expresses the panGABA markers *Ascl1*, *Gad1* and *Reelin* and rTh transcription factor genes *Gata2*, *Tal1*, *Sox14* (Figure 2B, C). Taken together, these data led us to conclude that ectopic expression of *Helt* in the cTh progenitor domain is sufficient to divert the fate of these progenitors from glutamatergic to GABAergic and more specifically towards a rTh GABAergic fate (Figure 2E). It should be noted that all rTh markers analyzed are also expressed by some GABAergic neurons in other brain compartments (for example, the caudal pretectum and the ventral and dorsal midbrain); it is, therefore, solely on the grounds that electroporated cells expressing *Helt* ectopically are contained within the thalamus that we conclude they have acquired a rTh identity.

pTh and thalamic domains are separated by the zli, a morphological and molecular landmark. We used expression of *Shh*, visualized by *in situ* hybridisation (ISH) on consecutive coronal brain sections (data not shown) to draw a dotted line dividing pTh and thalamic compartments (Figure 2A, B, D). In contrast to the effect seen in the thalamus, ectopic expression of *Helt* in the pTh failed to induce markers of rTh subtype identity (Figure 2D), implying that the activity of prepatterning genes, such as *Irx3*[8,13] may be required for the observed thalamic phenotype.

### Dlx2 induces prethalamic GABAergic neurogenesis in the thalamus

**Figure 3:**
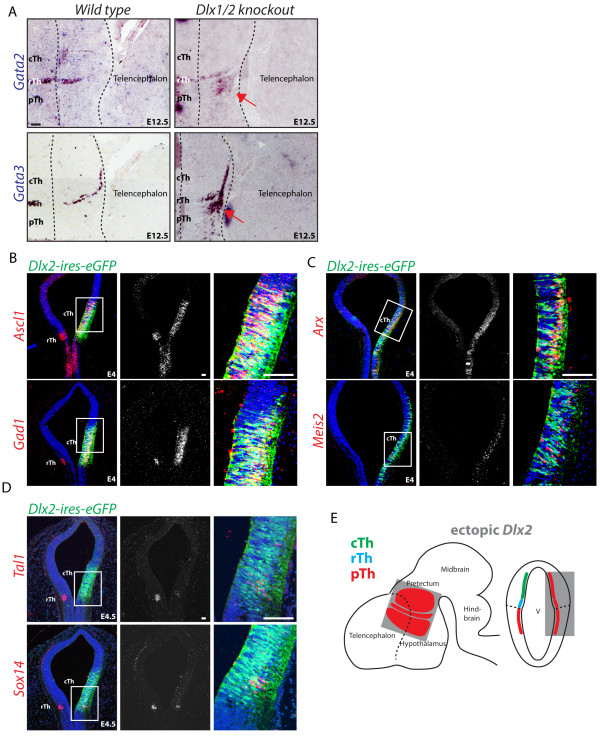
**Dlx2 induces GABAergic differentiation and suppresses rostral thalamic (rTh) subtype identity.** Key rTh transcription factor genes *Gata2 and Gata3* are expressed in the pTh of *Dlx1/2* compound knockout mice (red arrows), providing strong evidence that *Dlx1/2* are required to maintain pTh identity **(A)**. Ectopic expression of the pTh gene *Dlx2* in the glutamatergic cTh, induces GABAergic differentiation, visualized by induction of the panGABAergic markers *Gad1* and proneural gene *Ascl1***(B)**. Ectopic expression of *Dlx2* induces pTh GABA subtype progenitor markers in the rTh and cTh (*Arx*, *Meis2*) **(C)**. Consistent with a role in controlling GABA subtype identity in the thalamus, rTh markers (*Tal1*, *Sox14*) are downregulated upon ectopic expression of *Dlx2***(D)**. A schematic summary of the effect of ectopic *Dlx2* expression in the progenitor domains rTh and cTh **(E)**. Scale bars: 100 μm.

Expression of *Dlx2* defines neuronal progenitors in the pTh and is not expressed in the rTh. Dlx2 regulates GABA progenitor migration and differentiation in the telencephalon [22] and the prethalamus [5] but its role in lineage fate decisions in the diencephalon has received limited attention [23,24]. We have shown that in the absence of both *Dlx1* and *Dlx2* genes (*Dlx1/2* compound knockout), the mouse pTh acquires a rTh fate, leading to the formation of an ectopic intergeniculate leaflet (IGL) expressing *Npy*[6]. We also observed how the ectopic rTh that forms in the pTh of *Dlx1/2* compound mutant mice lacks expression of the earliest rTh marker *Helt*. This is supportive of a model whereby GABA progenitors retain latent plasticity for subtype identity upon cell cycle exit. The model finds further support in the recent report that *Gata2* and *Gata3* act post mitotically in the rTh to suppress the alternative pTh fate by suppressing *Dlx2* expression [18]. To test whether the reciprocal regulation is also true, we investigated *Gata2* and *Gata3* expression in the pTh of the *Dlx1/2* compound knockout mouse (Figure 3A). In agreement with our previous observation [6] the *Gata2 and Gata3* genes are also ectopically expressed in the pTh of *Dlx1/2* mice, further confirming that Dlx1/2 act as lineage identity factors to support a pTh GABA subtype fate. To further investigate the role of *Dlx2* in cell lineage decisions, we forced expression of *Dlx2* in the thalamus. Forced expression of *Dlx2* in the cTh induces a glutamatergic to GABAergic fate switch as shown by the induction of the proneural gene *Ascl1* and panGABA marker *Gad1* (Figure 3B, E); ectopic GABA differentiation driven by Dlx2 presents features of pTh identity (*Arx*, *Meis2*) (Figure 3C); this result is consistent with the previous observation of ectopic induction of the *Dlx1/2* effector gene *Arx* under similar overexpression conditions [5]. Ectopic *Dlx2* expression in the rTh does not alter its overall GABAergic fate, but suppresses rTh GABA subtype identity (*Tal1*, *Sox14*) (Figure 3B, D, E).

### Sox14 is required for rostral thalamic subtype marker expression

**Figure 4:**
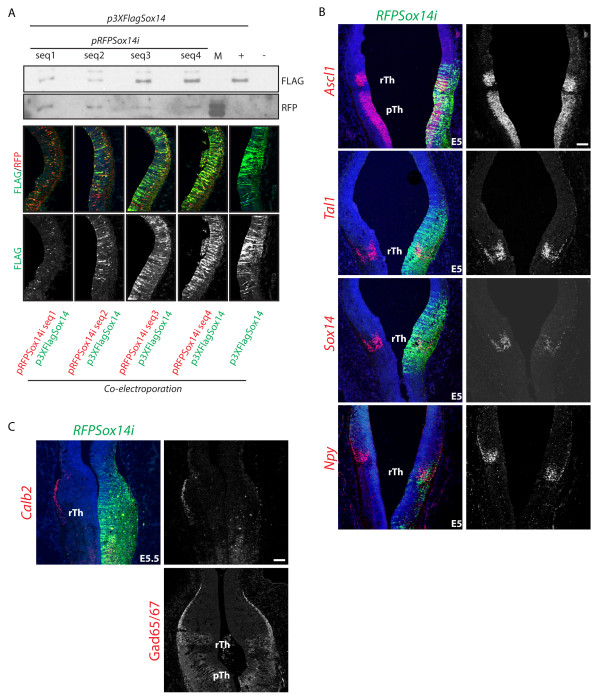
***Calb2 *subtype marker expression requires *Sox14.*** RNA interference was used to downregulate expression of the endogenous *Sox14* gene in the rTh. Four different sequences of the chick *Sox14* mRNA were targeted (seq1 to 4); their effect in inducing *Sox14* mRNA degradation was tested by transient co-transfection in cell culture or co-electroporation in the embryo. The Sox14i seq2 was used to obtain the data in **A** and **B(A)**. Acute downregulation of *Sox14* from E2.5 has no effect on the expression of rTh genes in the proliferative (*Ascl1*) and postmitotic (*Tal1, Npy*) domains, whilst a reduction of endogenous *Sox14* mRNA is observed **(B)**. Lack of *Calb2* expression in the rTh expressing a Sox14i construct. Despite the strong downregulation of the subtype marker *Calb2*, no changes in expression of the panGABAergic marker GAD65/67 could be detected; this, together with the lack of any detectable effect on other pan- and rTh GABAergic transcription factors suggests a restricted role for *Sox14* in controlling GABA neuron subtype specific gene expression **(C)**. Scale bars: 100 μm.

*Sox14* is expressed by GABAergic progenitors in the rTh upon cell cycle exit [6]. We described a *Sox14* loss of function phenotype in the developing mouse diencephalon, suggestive of a role in positioning of rTh derivatives in the developing thalamus [6]. A possible role for *Sox14* in regulating some aspects of subtype identity is supported by our previous observation that the proportion of rTh neurons expressing the GABA subtype marker *Npy* is increased in the embryonic *Sox14* knockout mouse, a phenotype that, we speculate, results from retention of ventral IGL derivatives within the presumptive IGL. Here, we have adopted a chick-specific RNA interference strategy, based on the U6 promoter expression of a microRNA operon [25], to achieved acute *Sox14* knockdown (*Sox14i*) in the developmental time window between E2.5 and E5.5 (Figure 4A). rTh progenitors with reduced *Sox14* expression retain their GABAergic fate, as indicated by the presence of the GABA enzymes Gad65/67 and expression of the rTh genes *Ascl1*, *Tal1* and *Npy* (Figure 4B), but fail to express the GABA subtype marker *Calb2* (Figure 4C).

**Figure 5:**
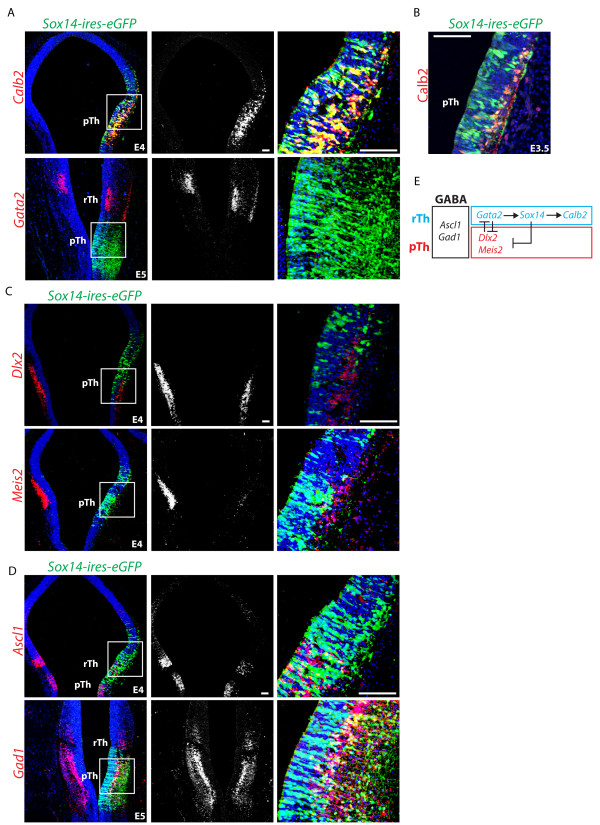
***Sox14 *expression induces *Calb2 *and suppresses prethalamic (pTh) GABA identity. ***Sox14* expression in pTh GABA progenitors results in the selective activation of the rTh subtype marker *Calb2,* whilst the upstream regulator of rTh identity *Gata2* was not activated **(A)**. Immunohistochemistry illustrating the co-localization of Calb2 protein in pTh progenitors expressing the transcription factor Sox14 **(B)**. Expression of pTh progenitor markers (*Dlx2* and *Meis2*) is reduced upon ectopic expression of *Sox14***(C)**, while the overall GABAergic identity is not affected (*Gad1*) **(D)**. Schematic recapitulation of the cross-regulation of GABA subtype restricted transcription factor on the rostral and pTh sides of the zli. Gata2/3 and Dlx1/2 have similar but reciprocal roles in suppressing the alternative subtype fate. Sox14, which acts downstream of Gata2 controls transcription of a subtype marker gene: *Calb2*. Its ectopic expression is not compatible with that of pTh transcription factor genes **(E)**. Scale bars: 100 μm.

To investigate further the relationship between *Sox14* and *Calb2*, we investigated the effects of ectopic expression of *Sox14* in the pTh *Calb2* negative GABAergic domain. Consistent with the observed loss of *Calb2* expression in the *Sox14i* rTh, we observed a rapid upregulation of *Calb2* in pTh GABA progenitors upon ectopic *Sox14* expression (Figure 5A). Ectopic Calb2 protein can be detected in the more mature neurons expressing ectopic *Sox14* but is absent in proliferating progenitors (Figure 5B), the delayed onset of expression is reminiscent of the late onset of *Calb2* expression seen in rTh-derived neurons (Figure 1C, D). *Gata2*, a rTh marker active upstream of *Sox14*[18] was not induced by ectopic *Sox14* (Figure 5A). Similarly, only a minor effect was noticed on the panGABA proneural gene *Ascl1* (Figure 5D), possibly due to the predicted function of *Sox14* in promoting cell cycle exit [26]. Yet, pTh genes *Dlx2* and *Meis2* were heavily downregulated upon *Sox14* expression (Figure 5C, E). Ectopic *Sox14* expression does not interfere with glutamatergic versus GABAergic fate determination in the cTh (not shown), nor does it interfere with general GABA differentiation (Figure 5D, E).

## Conclusions

In this article we describe the organization of neuronal progenitor domains in the chick thalamus on the basis of previous findings in the zebrafish and mouse and conclude that this is well conserved between the three model systems. Taking advantage of the conservation between early neurogenesis in the chick and mouse, we then used *in ovo* manipulations of gene expression to further investigate the presence of two alternative transcriptional programmes on both side of the mid-diencephalic organizer zli that guide neuronal progenitor differentiation down the GABAergic pathway, thus generating distinct thalamic inhibitory subtypes. The accessibility of the chick embryo and its amenability to *in vivo* manipulations facilitated the discovery of novel functions for three developmentally regulated transcription factors: *Helt*, *Dlx2* and *Sox14,* which contribute to specify GABAergic neuron diversity in the thalamus. Helt is sufficient to divert cTh progenitors towards a rTh GABA subtype fate. This function is likely to depend on the previous expression of prepatterning genes that confer thalamic identity over the pTh one, as *Helt* expression in the pTh does not change GABA subtype identity. In agreement with previous observations [5], we confirm that Dlx2 is sufficient to induce the GABAergic fate in excitatory thalamic progenitors, inducing expression of *Ascl1*, *Gad1*, *Meis2* and *Arx*. Furthermore Dlx2 can suppress the rTh GABA subtype fate via downregulation of *Tal1* and *Sox14*. Sox14 acts post mitotically to induce the rTh subtype marker and calcium binding protein calretinin (*Calb2*). *Calb2* induction by Sox14 is rapid, as it anticipates the time of normal expression in the rTh (E5.5), but follows nonetheless the stepwise progression along cellular differentiation as it is transcribed in *Sox14*-expressing pTh neurons in the mantel layer and not in those in the proliferative zone. At the same time, *Sox14* can repress other transcriptional regulators in the pTh, such as *Dlx2*, *Meis2* and *Arx*. These new findings add support to the emerging view that differentiating GABAergic neurons retain subtype lineage plasticity upon leaving the cell cycle: in the pTh, Dlx1/2 suppress a latent rTh fate [[6] and this manuscript], whilst in the rTh, Gata2/3 suppress a latent pTh fate [18]. In both cases, cells have already progressed from progenitors to precursors. Given that a similar transcription factor cascade to the one described for the rTh occurs also at other brain locations, it would seem reasonable to speculate that preexisting patterning genes and extrinsic factors play an essential role in shaping up the full transcriptional profile of otherwise similar GABAergic neurons in functionally distinct anatomical regions. The evolution of two alternative and mutually exclusive GABAergic differentiation programmes in the thalamus may be instrumental to achieve a broad spectrum of inhibitory synaptic connectivity and electrophysiological properties at this crucial anatomical node along corticopetal and corticofugal pathways. It should be considered that, whilst the organization of the main neuronal progenitor domains is conserved, different cell sorting and migratory pathways shape the adult thalamus in the different model systems, hence the main adult structure derived from rTh progenitors is the IGL in the mouse and the perirotundic area in the avian system [27,28]. Whilst we have further characterized the developing IGL as a source of tangentially migrating GABA neurons in the thalamus [6], it remains unknown whether the avian perirotundic area asserts a similar function. In consideration of the broad functional heterogeneity of GABA neurons in the thalamus, it is likely that transcriptional regulation continues to play a crucial role well after the initial differentiation of rTh and pTh GABA neurons, to further guide the acquisition of diverse cellular properties.

## Methods

### Chick and mouse embryos

Fertilized hens eggs (Henry Stewart, Louth, UK) were incubated in a humidified room at 38°C for 3 to 6 days before harvesting and dissecting in cold PBS for whole mount ISH. Staging of chicken embryos was according to the embryonic day (E) since egg incubation began. Fixed (4% PFA) double knockout *Dlx1/2* mouse embryos and littermate controls were a kind gift from John L Rubenstein (UCSF). Mice were bred and maintained at UCSF under local ethical and legal regulations.

### Gain and loss of function DNA vectors

To achieve a knockdown effect on the expression level of endogenous *Sox14*, we adopted the strategy described in [25]. Briefly, 22 nucleotide target sequences were identified using an siRNA design tool available at (http://sirna.wi.mit.edu) (Whitehead Institute for Biomedical Research) starting at positions: 299, 500, 545 and 745 of NCBI sequence NM_204761. *Sox14* specific oligonucleotides and a scrambled sequence were subsequently cloned in the second miRNA site of vector pRFPRNAi to generate synthetic miRNA30-like hairpins under the control of the chick U6 snRNA promoter, as described in [25].

To drive ectopic expression of *Helt,* a mouse cDNA containing the entire coding sequence (CDS) of the gene was cloned downstream of the constitutive promoter of the pCAGGS vector to transcribe a bicistronic mRNA containing *ires-eGFP*. Similarly, to drive expression of *Sox14*, the entire CDS of the chick gene was cloned in the pCAGGS-ires-eGFP vector. The CDS of chick *Sox14* was also cloned in frame with three FLAG tags in the p3XFLAG-CMV-7.1 (Sigma-Aldrich, St.Louis, Missuouri, USA) to generate an N-terminus FLAG-tag version of the protein. Ectopic expression of mouse *Dlx2* was obtained using a constitutive expression vector (pCAGGS) kindly provided by John L Rubenstein.

### Evaluation of gene knockdown efficiency

In order to assess which of the four pRFPSox14i plasmids had the strongest knockdown effect, each pRFPSox14i DNA and an equimolar amount of p3XFLAGSox14 was co-transfected in cultured COS cells using a standard Lipofectamine protocol (Thermo Fisher Scientific, Massachusetts, USA). Total cell lysate for each *Sox14i* combination was diluted 400 times and used as substrate to detect FLAG and RFP proteins by Western blotting (mouse anti-FLAG Sigma F1804; used at a dilution of 1:3,000) and rabbit anti-RFP (Millipore, Massachusetts, USA AB3216; used at a dilution of 1:500). To test for downregulation *in vivo*, each pRFPSox14i vector in equimolar combination with the p3XFLAGSox14 was co-electroporated in the diencephalon of E2.5 chick embryos. Electroporated embryos were collected after 48 hours of incubation and processed for immunohistochemical detection with mouse anti-FLAG (Sigma F1804; used at a dilution of 1:1,000) and rabbit anti-RFP (Millipore, Massachusetts, USA AB3216; used at a dilution of 1:100) on coronal sections.

### *In ovo* electroporation

Chick embyros were electroporated at E2.5 using fine platinum electrodes and an ElectroSquare Porator, Harvard Apparatus, Massachusetts, USA. Settings were as follows; 15 V, 3 pulses, 50 ms pulse duration, 950 ms interval. DNA was diluted to a final concentration of 1 μg/ml and fast green added to aid visualization. DNA injected into the neural tube at diencephalon level; electrodes were placed either side of the diencephalon over the thalamus or prethalamus. DNA overexpression vectors plasmids used; pCAGGS-Helt-ires-eGFP, pCAGGS-Sox14-ires-eGFP, pCAGGS-Dlx2 (gift from John L Rubenstein, UCSF) (co-electroporated with pCAGGS-eGFP), pRFPSox14i(1-4), pRFPscrambled and p3XFLAGSox14.

### *In situ* hybridisation and immunohistochemistry

Electroporated and control embryos were dissected in ice-cold PBS and cryoprotected by passage through serial dilutions of sucrose/PBS equilibration; 10%, 20% and finally 30% sucrose/PBS. Samples were then embedded (Tissue Tek, Sakura, Torrance, Canada) and frozen by floating on liquid nitrogen. Cryosections were cut at 12-μm thickness. ISH was carried out as published in [29]. In brief: hybridisation with digoxigenin (DIG) and or fluorescein isothiocyanate (FITC; Roche, Basel, Switzerland) labeled riboprobes was carried out overnight at 65°C. Washing steps were carried out in MABT (100 mM maleic acid, 150 mM NaCl, 0.1% Tween 20). Blocking was performed in 2% Boehringer Blocking Reagent (BBR) (Roche, Basel, Switzerland), 20% normal goat serum in MABT. The anti-DIG or anti-FITC alkaline phosphatase (AP)-conjugated antibody (Roche, Basel, Switzerland) was incubated overnight at 4°C. Color reaction was developed using NBT/BCIP substrate in NTMT (pH 9.5) or Fast Red in Tris (pH 8.2). For two color ISH, the first AP-conjugated antibody was removed from the section via a 30-minute incubation in 5 mM EDTA/PBS at 70°C. Chick anti-Gata2 ISH probe was a gift from Anthony Graham (KCL). Mouse anti-Gata2/3 ISH probes were a kind gift from Maxim Bouchard (McGill University). After completion of ISH protocol, anti-GFP immunohistochemistry was carried out in order to identify electroporated cells. Sections were blocked in 5% normal goat serum with 0.25% Triton X-100 for one hour at room temperature, rabbit anti-GFP (Thermo Fisher Scientific, Massachusetts, USA A11122, 1:100 dilution) added to block incubated overnight at 4°C, secondary antibody goat anti-rabbit Alexa488 (Thermo Fisher Scientific, Massachusetts, USA; added to block at a dilution of 1:500), incubated for 1 hour at room temperature. Other antibodies used were: guinea pig anti-Sox14 (gift from Thomas Jessell, Columbia University, New York; used at a dilution of 1:5,000), rabbit anti-calretinin (Calb2, AbCam, Massachusetts, USA ab702; used at a dilution of 1:100), rabbit anti-Gad65/67 (AbCam, Massachusetts, USA ab11070; used at a dilution of 1:5,000) rabbit anti-RFP (Millipore, Massachusetts, USA AB3216; used at a dilution of 1:100) and goat secondary Alexa-conjugated antibodies (Thermo Fisher Scientific, Massachusetts, USA; used at a dilution of 1:500). The sections were then nuclear counterstained with 100 ng/ml Hoechst in PBS for 10 minutes before mounting in antifade Prolong Gold (Thermo Fisher Scientific, Massachusetts, USA). Images were taken with an Eclipse series Nikon, Tokyo, Japan confocal microscope and processed with Image J (rsbweb.nih.gov/ij/).

## Abbreviations

AP: alkaline phosphatase; BBR: Boehringer Blocking Reagent; CDS: coding sequence; cTh: caudal thalamus; DIG: digoxigenin; FITC: fluorescein isothiocyanate; GABA: γ-aminobutyric acid; IGL: intergeniculate leaflet; ISH: *in situ* hybridisation; nonTRN: GABAergic neurons in the thalamus excluding the TRN; PBS: phosphate-buffered saline; pTh: prethalamus; rTh: rostral thalamus; TRN: thalamic reticular nucleus; zli: zona limitans intrathalamica.

## Competing interests

The authors declare that they have no competing interests.

## Authors’ contributions

KS, VZ and AD carried out experiments and acquired images; AGL and AD provided financial support for the project; AD, KS and AGL designed experiments; AD wrote the manuscript. All authors read and approved the manuscript.
